# A new paradigm for clinical communication: critical review of literature in cancer care

**DOI:** 10.1111/medu.13204

**Published:** 2016-12-20

**Authors:** Peter Salmon, Bridget Young

**Affiliations:** ^1^ Department of Psychological SciencesUniversity of LiverpoolLiverpoolUK

## Abstract

**Objectives:**

To: (i) identify key assumptions of the scientific ‘paradigm’ that shapes clinical communication research and education in cancer care; (ii) show that, as general rules, these do not match patients’ own priorities for communication; and (iii) suggest how the paradigm might change to reflect evidence better and thereby serve patients better.

**Methods:**

A critical review, focusing on cancer care. We identified assumptions about patients’ and clinicians’ roles in recent position and policy statements. We examined these in light of research evidence, focusing on inductive research that has not itself been constrained by those assumptions, and considering the institutionalised interests that the assumptions might serve.

**Results:**

The current paradigm constructs patients simultaneously as needy (requiring clinicians’ explicit emotional support) and robust (seeking information and autonomy in decision making). Evidence indicates, however, that patients generally value clinicians who emphasise expert clinical care rather than counselling, and who lead decision making. In denoting communication as a technical skill, the paradigm constructs clinicians as technicians; however, communication cannot be reduced to technical skills, and teaching clinicians ‘communication skills’ has not clearly benefited patients. The current paradigm is therefore defined by assumptions that that have not arisen from evidence. A paradigm for clinical communication that makes its starting point the roles that mortal illness gives patients and clinicians would emphasise patients’ vulnerability and clinicians’ goal‐directed expertise. Attachment theory provides a knowledge base to inform both research and education.

**Conclusions:**

Researchers will need to be alert to political interests that seek to mould patients into ‘consumers’, and to professional interests that seek to add explicit psychological dimensions to clinicians’ roles. New approaches to education will be needed to support clinicians’ curiosity and goal‐directed judgement in applying this knowledge. The test for the new paradigm will be whether the research and education it promotes benefit patients.

## Introduction

Social scientists’ collaboration with clinicians over three decades has shown that good communication underlies effective health care,^1^ has established communication curricula in clinical training,^2^ and has generated guidance about how to communicate with patients across clinical situations from meeting new patients to breaking bad news.^3^, ^4^ In particular, several decades of research and associated curriculum and policy development have embedded communication training and guidance in cancer care internationally,^5^, ^6^, ^7^, ^8^, ^9^, ^10^ with curricula sponsored by governments and major health providers being widely disseminated to cancer clinicians.^11^, ^12^, ^13^ Nevertheless, recent reviews find little evidence that cancer patients benefit after clinicians are taught communication.^9^, ^14^, ^15^, ^16^ Although training can change clinicians’ communication, for instance by increasing open questions or empathic statements, effects on patients’ satisfaction, well‐being or clinical outcomes have proved elusive. The reviews’ authors recommend improved research designs in a continued effort to show that training does help patients. However, there are concerns that expert guidance on communication is often unrealistic,^17^, ^18^, ^19^, ^20^, ^21^ and many clinicians and students remain sceptical of it.^11^, ^20^, ^22^, ^23^, ^24^, ^25^, ^26^, ^27^, ^28^, ^29^, ^30^, ^31^ Moreover, social scientists have challenged assumptions on which communication education and guidance in cancer and across health care are based.^32^, ^33^, ^34^ In this context, a more radical response to negative findings about the effects of communication education is to reappraise the direction the field has taken. Reappraisal could directly benefit the large and growing population of patients in cancer care, and might also offer lessons for the broader field of clinical communication.

In science, theory directs what researchers study and educators teach. Some of this is explicit in formal theories, but more pervasive and influential assumptions shared by communities of scientists are implicit as ‘paradigms’; that is beliefs, often unacknowledged, that shape scientists’ choices of subjects, methods and explicit theories.^35^ Paradigmatic assumptions become particularly embedded in social science because they acquire normative dimensions; that is, they specify how people ‘should be’ rather than just describing how they ‘are’.^33^, ^36^ Previous writers have warned of the interweaving of normative assumptions with scientific theory in clinical communication.^2^, ^37^ Our aims here are to: (i) identify key assumptions of the ‘paradigm’ that has shaped clinical communication research and education in cancer care; (ii) show that, as general rules, these do not match patients’ own priorities for communication in practice; and (iii) suggest how the paradigm might change to reflect the evidence better and thereby serve patients better.

A new paradigm will not arise from systematic reviews of research literature. By aggregating literature to answer specific questions, in particular ‘what works’, systematic reviews tend to perpetuate the paradigm that gives rise to those questions.^38^ They are not well suited to questioning the assumptions that led to those questions. Instead we adopted an approach, described by some methodologists as an ‘interpretive’ or ‘critical’ review,^39^, ^40^ which aims to produce new ideas rather than answer specific questions. This kind of review emphasises the conceptual contribution of selected items of literature, rather than procedures of comprehensive search and synthesis. It critiques research findings and the paradigms underlying them, and is creative in offering new ideas rather than aggregating literature.^41^ Reviews of this kind have been important in questioning assumptions that shape communication research and guidance.^18^, ^32^, ^33^, ^42^

As our starting point, we drew on recent position statements in academic journals and health service guidance for practitioners drawn from the UK, Europe, the USA and Australia^6^, ^7^, ^8^, ^9^, ^12^, ^13^, ^43^, ^44^ to identify defining assumptions of the current paradigm. We compared these assumptions with research evidence of patients’ own priorities for clinical communication and evidence of clinicians’ views about how they meet patients’ communication needs. Whereas most research has used questionnaires and observational instruments that measure what researchers believe is important, even though this is not necessarily what matters to patients,^45^, ^46^ we selected research that was inductive (i.e. that sought to generate new theory from detailed observations). This meant focusing on research using qualitative methods because these try to minimise the influence of researchers’ preconceptions on the research process.^47^, ^48^ Because a critical review is intended not to aggregate literature but to develop new ideas, it requires a purposive rather than systematic approach to identifying literature.^39^, ^40^ That is, literature is selected for its ability to inform conceptual development. Qualitative literature is notoriously hard to search, reflecting the lack of defining methodological terms.^49^, ^50^ Therefore we used a range of methods. We drew initially from our own programme of critical inductive research in cancer communication and from those of other authors of whom we were aware, supplemented by search of the Science and Social Science Citation Indices using key words ‘cancer’, ‘qualitative’ and ‘communication’, and by examining bibliographies of identified papers until we reached theoretical saturation (i.e. additional papers were no longer contributing information that changed the analysis).^40^ Individually, the authors identified potentially relevant papers and both authors read and discussed each one. Like other authors of critical reviews, we do not claim that our review is systematic or reproducible. Rather, we provide an account, grounded in inductive literature, the utility of which educators and researchers can judge through debating and applying the ideas we provide.

## Patients: vulnerable and dependent

Two defining elements of the current paradigm are salient in position statements and guidance about patients’ communication needs. First is the assumption that patients are fundamentally autonomous, in the sense that they are responsible for decisions about their own care.^51^ This assumption, visible in the extensive guidance that focuses clinicians on informing patients about illness and treatment and on empowering them to join in treatment decisions,^6^, ^7^, ^9^, ^13^, ^43^, ^44^ is normative in specifying how clinicians *should* protect patients’ autonomy.^2^ It also drives research on how clinicians *can* identify and address patients’ information needs and help them make treatment decisions. The second element of the paradigm is the assumption that patients are emotionally needy and look to clinicians for support, and this is visible in guidance on clinicians engaging with patients’ emotional cues and needs.^6^, ^7^, ^9^, ^12^, ^43^ This assumption, too, is normative in evoking expectations of compassion in practitioners, while also driving research into the processes and outcomes of their emotional engagement with patients.^52^, ^53^, ^54^

These two assumptions, depicting patients as self‐determining in clinical relationships, but also emotionally needy, seem contradictory. Moreover neither, as a general rule, fits the inductive evidence about patients’ needs. First, inductive studies of what patients and families seek from consultations where stakes are high do not support the overwhelming emphasis on patients as consumers of information or as decision makers. Patients and families have diverse needs that change over time,^55^, ^56^, ^57^, ^58^ but findings consistently emphasise their need to feel cared for, to hope for the future and to trust their clinicians’ decisions and recommendations. Information is valued where it enables hope and sustains trust,^55^, ^57^, ^59^, ^60^, ^61^, ^62^ so patients need doctors to manage information carefully and often to constrain and pace it.^56^, ^57^, ^63^, ^64^, ^65^ Similarly, the desire to be decision makers is not prominent, patients typically preferring to trust doctors’ recommendations^55^, ^57^, ^66^, ^67^, ^68^, ^69^, ^70^, ^71^, ^72^ provided the doctor gives sufficient reason.^63^, ^64^, ^73^, ^74^ Patients can still feel autonomous while relying on clinicians; that is, autonomy is relational and arises from trusting clinicians’ care and expertise and feeling respected as an individual.^55^, ^75^ The second element of the current paradigm, that patients are emotionally needy, also compares poorly with inductive evidence. Although qualitative research exposes patients’ and families’ despair and fear, many patients (or, where patients are children, their parents) prefer to avoid explicit emotional talk with practitioners. Instead, they can gain comfort from doctors prioritising clinical care over counselling or from nurses talking about daily life rather than emotional feelings.^63^, ^76^, ^77^, ^78^, ^79^ From patients’ perspectives, therefore, asymmetry of vulnerability and clinical expertise is fundamental to clinical relationships where they are in mortal danger, such as in cancer care: patients are vulnerable and depend on clinicians’ expertise and authority. This asymmetry has resisted decades of effort to reduce it by communication practices, indicating that it is integral to clinical relationships and should therefore define the paradigm of communication education and research.^32^

## Clinicians: goal‐directed experts

The concept of ‘communication skills’ dominates communication experts’ expectations of clinicians. It reflects a belief that communication can be divided into discrete ‘skills’, ranging from basic elements of interpersonal behaviour such as ‘eye contact’ to psychological qualities such as ‘empathy’. Psychological skills are said to equip clinicians to build relationships and provide the emotional engagement that patients need.^80^, ^81^ Clinicians are widely criticised for lacking skills and admonished to learn them.^6^, ^7^, ^8^, ^9^, ^82^ Published lists and definitions of communication skills, curriculum guidelines and educational and assessment techniques help educators teach these skills.^11^, ^12^, ^13^ In depicting communication as a set of skills, with standardised procedures for applying and teaching them, the current paradigm constructs communication as a ‘health technology’^83^ and clinicians, correspondingly, as communication technicians.

Students and practitioners sometimes complain of feeling ‘deskilled’ by expectations to learn and perform communication skills.^22^ Moreover, clinicians, educationists and social scientists have warned over three decades that the concept of skills misrepresents communication.^18^, ^84^, ^85^ For instance, when behaviours, such as making eye contact or talking empathically, are denoted as ‘skills’, the implication is that they are inherently and consistently beneficial. In reality, however, the meaning of communication behaviour usually depends on the context: eye contact can be threatening and patients can experience psychosocial talk as intrusive or inappropriate.^76^, ^77^, ^79^, ^86^ This explains why patients’ experiences of a clinical relationship are not closely related to the communication ‘skills’ that clinicians perform.^45^, ^63^, ^78^ Therefore communication researchers and educators oversimplify their subject and misrepresent patients’ needs when they seek to standardise communication in consultations,^31^ or when they focus on how often a ‘skill’ is used, regardless of context, for example in criticising clinicians for showing too little empathy or exhorting them to learn to display more.^54^, ^87^

Researchers have unfortunately not been very concerned with clinicians’ views about how they try to meet patients’ communication needs. However, in a recent study breast cancer surgeons described communication goals and strategies that explained why they often did not follow formal guidance,^28^, ^63^ just as previous studies found in primary care physicians.^88^, ^89^ For instance, surgeons avoided detailed prognostic information where their priority was to strengthen patients’ morale.^28^ Unsurprisingly, the surgeons described gaining little from communication skills training, preferring to observe experienced colleagues and to reflect on their own practice. Some educational programmes prescribe sets of communication goals, or ‘tasks’, such as ‘elicit information’ or ‘understand the patient's perspective’, instead of skills.^90^ However, pre‐specified tasks might not suit all consultations, and clinicians might have good reason to prioritise others. Moreover, what works for one practitioner might not for another.^91^ Given, too, that the ‘chemistry’ of dyadic relationships is notoriously unpredictable, and that only the participants will know much of the unique context for any instance of communication, experts’ general rules, whether about tasks or skills, inevitably have limited purchase.^19^, ^85^

The cancer communication literature, like communication literature more broadly, focuses on skills and tasks to the exclusion of goals and outcomes.^92^ To engage more effectively with clinicians, communication education and research would need to make their communication goals the starting point. Good communication would be promoted, not by expecting clinicians to follow rules but by enhancing the quality of their judgements about what their goals should be and how to achieve them.^93^, ^94^ Bringing clinicians’ judgements about goals to the foreground of communication education, where they can be respected or challenged, offers a potentially more realistic way to influence their communication than starting with the assumption that they just need to learn skills.^94^

## Theory for a new paradigm

A new paradigm needs more than recognition of the fundamental asymmetry of clinical relationships in cancer care and the goal‐directed nature of clinicians' communication. It needs formal theory that can be a lens for researchers and educators to examine clinical communication and focus on new questions and approaches that engage with this asymmetry and goal‐directedness. Recognising the need to place patients’ vulnerability at the centre of their work, some educators and researchers have approached clinical relationships from the perspective of attachment theory.^95^, ^96^, ^97^, ^98^ This theory centres on the need to feel safe in the face of threat and explains the intense emotional bonds that people can form with those whom they imbue with power to protect them. First applied to parent–infant relationships,^99^, ^100^ it has been extended to romantic relationships and friendships in adults as well as clinical relationships.^101^, ^102^, ^103^ There are, of course, important differences between adult and child attachments.^104^, ^105^, ^106^ An infant can be reassured by proximity to an emotionally comforting caregiver, but adults are more likely to need evidence that a caregiver can, in fact, protect them. Adults can make use of symbolic representations of attachment figures, for example anticipating an impending meeting or knowing that they can contact the attachment figure if they need to, rather than needing physical proximity.^107^ For adults, security can even mean appropriate distance and separation, rather than intimacy.^105^ Nonetheless, patients’ relationships with clinicians, particularly when life is at stake such as in cancer care, can resemble attachment, inasmuch as patients build an image of their clinician centred on the clinician's expertise and authority and shaped by their own attachment needs, and see clinicians and the systems in which they operate as providing a ‘safe haven’ in the face of threat.^60^, ^62^, ^97^, ^108^, ^109^, ^110^

Because clinicians’ authority underpins the clinical relationship, attachment theory warns that positioning patients as ‘partners’ and decision makers will usually be a distorting lens for understanding their experience of care. Indeed, the theory indicates that patients’ sense of control in the face of illness arises directly from being able to depend on the ‘secure base’ that clinicians and the health care system can provide.^70^, ^109^, ^111^ Attachment theory is mirrored in recent bioethics ideas of relational autonomy, according to which patients’ autonomy lies, not in having treatment choices, but in a sense of relationship with expert clinicians and in feeling confident about clinical decisions. Some patients, in some situations, will gain confidence from having made decisions themselves but most will want to trust clinicians’ recommendations; that is, to ‘own’ these recommendations and feel committed to them.^73^, ^112^, ^113^, ^114^ This, in turn, points to the need for clinicians skilled in explaining why they have recommended options and in checking whether patients are content with them.^73^, ^115^

Similarly, whereas communication literature currently urges clinicians to engage patients in emotional discussion to comfort them, attachment theory reminds us that the key to comforting patients is helping them feel safe.^95^ Therefore, it explains why cancer patients and their families appreciate clinicians who are calm, confident and authoritative rather than engaging in emotional talk.^63^, ^74^, ^76^ It explains why clinical relationships in cancer need not be ‘built’ by clinicians’ communication but can be present from the start in patients’ minds, arising from their own dependence and the clinicians’ expertise.^37^, ^60^, ^116^

Of course, patients differ in what they need from clinicians to help them be autonomous and feel safe, and their needs evolve over time.^68^ However, attachment theory frees researchers and educators from the ‘one‐size‐fits‐all’ character of current communication theory. For instance, the conceptual framework of ‘attachment styles’ helps to understand how life experiences can damage people's ability to trust clinicians and why different people need different things from clinical relationships.^117^, ^118^, ^119^ It might also help to understand practitioners’ own relationship styles.^95^

Crucially, despite its origin in studies of parenting, attachment theory is not paternalism repackaged. Medical paternalism is dangerous because it slips into the lazy assumption that ‘clinicians always know best’. Attachment theory emphasises the centrality of clinicians’ authority and expertise in meeting patients’ needs, not in deciding what those needs are.

## Implications for education: teaching knowledge and stimulating curiosity

This critical review points to defining aspects of a new paradigm within which educators can examine and debate what they do. Identifying the educational goals and methods that will develop and implement the paradigm will be a big challenge and our review does not aim to specify these. Nevertheless we can point to some ways in which a curriculum might change.

Our proposal would switch educators’ primary target from clinicians’ skills to the quality of their judgements about goals and how to reach them. This shift would instigate profound educational changes. Education to support clinicians’ judgements about other aspects of clinical care is founded on teaching knowledge. Doctors are expected to make judgements about pharmacotherapeutics, not by just following prescribing guidelines, but by applying fundamental knowledge of physiology and pharmacology. Focusing on skills has distracted educators from teaching knowledge about relationships as the foundation for clinicians’ communication. After all, a technician needs to know the skills to deploy, not the science behind them.

A communication curriculum could therefore start with knowledge about human relationships, particularly when one party is vulnerable. Learning about attachment processes and adult attachment styles could help clinicians make sense of the variability of patients’ presentations and appreciate, for example, that some patients’ difficulties with trust can lead to detachment or hostility that is easily mistaken for self‐sufficiency.^96^, ^97^ Knowledge about relationships will provide the foundation for more practically‐focused learning. As in other areas of clinical practice, good communication needs clinicians to transcend generic knowledge and be curious about their patients; that is, to be motivated to find out about their patients’ individuality^31^, ^120^ Emphasising skills might detract from this curiosity by focusing clinicians’ attention on their own behaviour instead of the patient or by promoting standardisation rather than sensitivity to patients’ individuality.^31^, ^121^, ^122^, ^123^ Cancer surgeons described learning by being mindful and reflective in communication with patients,^28^ and several educationists have proposed methods based on mindfulness or reflection as ways to facilitate curiosity and attentiveness to patients.^31^, ^120^, ^124^, ^125^, ^126^ Such approaches have the important strength that they can exploit the ‘tacit’ knowledge that clinicians already have by virtue of their own practice.^127^

In prioritising knowledge and curiosity, classroom and role‐play methods that communication educators already use will remain helpful. However, educators will need to exchange an underlying pedagogy based on inculcating pre‐specified skills for one that respects and supports clinicians’ role in fashioning and judging responses to the situations they encounter in practice. This will bring considerable challenges, but existing literature already suggests possibilities. We and others proposed that educators could learn from creative arts in encouraging creativity and judgement, with educators as critical ‘connoisseurs’.^19^, ^85^, ^128^, ^129^ Educators will need attitudinal change, too. Because the meaning of communication lies not in observable behaviour but in how this is experienced, because experience is highly context‐dependent and because clinicians have to make goal‐directed judgements in light of contextual information that often only they will know, experts will need to restrain criticism of clinicians’ communication. They will themselves need to show curiosity in understanding and studying why clinicians communicate as they do.

## Implications for research: the importance of ‘practice‐based evidence’

Because the defining features of the present paradigm have not arisen from research evidence, we must be alert to other forces that shape assumptions in this field so that we can guard against them. How social science depicts patients and their relationships with health care is notoriously susceptible to broader cultural and political interests^130^ and, in clinical communication specifically, several writers have suggested that assumptions that define the current paradigm have been shaped in this way.^2^, ^32^, ^33^, ^37^ Educators and researchers serve political agendas around individual responsibility and challenging professional power when they promote modes of communication that subserve patient empowerment.^2^, ^33^ In reducing an intangible area of clinical care to ‘skills’ that can be taught and measured, their emphasis on communication skills reflects a broader cultural belief in the power of technology to solve human problems.^36^, ^85^ There are professional interests at stake too. Psychiatrists and psychologists pioneered communication education and research, particularly in cancer.^131^, ^132^ Three decades later, a cadre of psychologically‐minded practitioners sustains communication teaching and research globally. Their professional interests lie in showing that clinicians are insufficiently psychological and can be taught to be more so. In clinical training, the language of ‘skills’ helped educators integrate communication into curricula that are widely regarded as skills based. Now, the concept of communication skills supports a burgeoning technology of methods to teach and assess communication, and the ‘experts’ who control this technology. It seems that much communication research, in effect, serves these interests rather than necessarily addressing patients’ needs; for instance, papers continue to report that educational programmes increase clinicians’ communication skills regardless of whether those skills benefit patients.^4^, ^19^ The effect of this kind of research is to entrench dominant frameworks without challenging them.

To inform the present critique we sought evidence particularly from inductive, qualitative studies of what patients and clinicians seek and do in communication in practice. Prioritising research of this kind will help ensure that the clinical communication enterprise is grounded in patients’ needs rather than in the broader cultural and professional interests that can shape deductive research.^46^

## Limitations of this review

As authors of this paper, we also are involved in the field, and this investment is simultaneously a strength and weakness of our review; our suggestions for theory for a new paradigm inevitably reflect our own experience and priorities. However, clinical communication needs to avoid theoretical hegemony, whereby a single framework comes to define the field and exclude competing views. Theories must be recognised as imperfect metaphors for making sense of the complexity of clinical communication rather than be applied as rigid templates or asserted as statements of dogma. Therefore we offer our ideas as contributions to the theoretical challenge and debate that the field needs if it is to be a genuinely scientific one, and not in an effort to supplant one theoretical hegemony by another. Attachment theory and ideas of goal‐directed practice provide a starting point for engaging with patients who feel vulnerable and doctors who want to help them. But limitations of both are already apparent. In clinical care, attachment relationships have to be asymmetric (the clinician cannot be as emotionally involved as the patient) and clinicians cannot be ‘non‐substitutable’ in the way that attachment figures in parental or romantic relationships are.^106^ Patients can feel protected by an efficient health care system and not just individual attachment figures within it.^109^ Viewing clinicians as goal directed leaves educators and researchers with considerable challenges in facilitating and changing clinicians’ goals and we can only provide pointers here. Theory will have to evolve, and educators and researchers will need to bring other theoretical insights to the mix.

Our review has focused on cancer care and we cannot generalise our proposals to other clinical specialties. However, our analysis hinges on two features that define patient and clinician roles in health care more broadly: patients’ vulnerability and clinicians’ expertise. Therefore researchers and educators can examine whether our analysis is helpful in other areas of care where patients who feel vulnerable seek help from expert clinicians.

## Conclusion

Drawing primarily on cancer care, we have argued that currently dominant elements of the clinical communication paradigm are not evidence based and lead researchers and educators to mould patients to cultural norms and political and professional interests. A new paradigm would make the reality of the clinical relationship the starting point for research and education to benefit patients. Adopting a new paradigm does not mean abandoning the endeavour to improve patients’ care and align it better with ethical norms. Instead, we argue that communication research and education are more likely to make a difference to health care if they take as their starting points the reality of what it means to be a patient or clinician in the context of mortal illness.

## Contributions

The authors jointly developed the ideas in this article and jointly prepared the manuscript. Both authors have approved the final manuscript.

## Funding

None.

## Conflicts of interest

None.

## Ethical approval

Not applicable.
